# LiveWell RERC State of the Science Conference Report on ICT Access to Support Community Living, Health and Function for People with Disabilities

**DOI:** 10.3390/ijerph17010274

**Published:** 2019-12-30

**Authors:** John Morris, Mike Jones, Frank DeRuyter, David Putrino, Catherine E. Lang, Danielle Jake-Schoffman

**Affiliations:** 1 Virginia C. Crawford Research Institute, Shepherd Center, Atlanta, GA 30309, USA; mike.jones@shepherd.org; 2 Department of Surgery, Duke University Medical Center, Durham, NC 27708, USA; frank.deruyter@duke.edu; 3 Department of Rehabilitation Medicine, Icahn School of Medicine at Mount Sinai, New York, NY 10029, USA; david.putrino@mountsinai.org; 4 Washington University School of Medicine, Saint Louis, MO 63108, USA; langc@wustl.edu; 5 Department of Health Education and Behavior, University of Florida, Gainesville, FL 32611, USA; djakeschoffman@ufl.edu

**Keywords:** mobile health, mHealth, mRehab, disability, rehabilitation, information and communication technology, accessibility, community participation, health and function

## Abstract

This article summarizes the proceedings of the three session State of the Science (SOS) Conference that was conducted by the Rehabilitation Engineering Research Center for Community Living, Health and Function (LiveWell RERC) in June 2019 in Toronto, Canada. RERCs customarily convene an SOS conference toward the end of their five-year funding cycle in order to assess the current state and identify potential future research, development, and knowledge translation efforts needed to advance their field. The first two sessions focused on the current and future state of information and communication technology (ICT) for mobile health (mHealth) and mobile rehabilitation (mRehab). The third session was a wide-ranging discussion of pressing needs for future research and development in the field. Several “big ideas” resulted from the discussion among participants in the SOS Conference that should inform the structure and operation of future efforts, including: (1) identifying active ingredients of interventions, (2) incorporating effective behavior-change techniques into all interventions, (3) including measures of social determinants of health in evaluation studies, (4) incorporating user-customizable features into technology solutions, and (5) ensuring “discoverability” of research and development outputs by stakeholders via structured and continuous outreach, education and training. Substantive areas of work include gaming and esports, the gamification of interventions for health and fitness, the cultivation of community supports, and continuous outreach and education wherever a person with a disability may live.

## 1. Introduction

This is the report on the proceedings of the State of the Science (SOS) Conference conducted by the Rehabilitation Engineering Research Center for Community Living, Health and Function (LiveWell RERC) in June 2019 in Toronto, Canada. Established in 2015, the LiveWell RERC is a partnership between Shepherd Center in Atlanta, Georgia; Duke University in Durham, North Carolina; and Northeastern University in Boston, Massachusetts. It is funded by the National Institute on Disability, Independent Living, and Rehabilitation Research (NIDILRR) of the United States Department of Health and Human Services (HHS). The LiveWell RERC was formed to examine access to information and communications technology (ICT) that could support independent living and community participation for people with disabilities.

NIDILRR-funded rehabilitation engineering research centers (RERCs) conduct advanced engineering research and develop innovative technologies that are designed to solve rehabilitation problems or to remove environmental barriers within a specific area of focus. RERCs also demonstrate and evaluate such technologies, facilitate changes to service delivery systems, stimulate the production and distribution of new technologies and equipment in the private sector, and provide training services.

The LiveWell RERC has a twofold mission: (1) provide ICT access to existing and emerging technologies for all people, regardless of ability, and (2) to develop and validate ICT applications that could support the independent living and community participation of people with disabilities. Early in the funding cycle, it became very clear through surveys of our 1500-member Consumer Advisory Network that the greatest interest related to ICT access was within the mobile healthcare (mHealth) and mobile rehabilitation (mRehab) space. Consequently, our work and our SOS conference focused on these areas of research and development.

## 2. Materials and Methods

It is customary for RERCs to convene a state-of-the-science conference toward the end of their five-year funding cycle in order to assess the current state of the field in their area of focus and identify potential future research, development, and knowledge translation efforts that are needed to advance the field. On 25–26 June 2019, the LiveWell RERC hosted a State of the Science Conference to set the agenda for future ICT research and development to promote the full inclusion of people with disabilities in the digital health revolution. The conference was held in collaboration with the American Congress of Rehabilitation Medicine (ACRM) and the Rehabilitation Engineering and Assistive Technology Society of North America (RESNA) as part of the cross disciplinary RehabWeek 2019 international conference in Toronto, Canada. This article summarizes the findings from the three-session SOS Conference. The first two sessions consisted of presentations and discussion by invited panelists—representing rehabilitation research, technology and consumer stakeholders—on topics related to the current and future state of ICT for mobile health (mHealth) and mobile rehabilitation (mRehab). The third session was a wide-ranging discussion among invited panelists and audience participants to help identify the most pressing needs and shape the agenda for future research and development in the field.

## 3. Results

### 3.1. Session 1: The Future of mHealth for People with Disabilities

- Introduction to mHealth for People with Disabilities:
  Frank DeRuyter, PhD, MMCi, Duke University Medical Center
- Capturing remote patient data and making it useful for clinicians:
  Devin Mann, MD, NYU Langone Health
- Technology adoption, demonstrating value to healthcare delivery organizations, clinicians and patients:
  David Putrino, PT, PhD, Mt. Sinai Medical Center
- Consumer perspectives, user acceptance/adherence, and abandonment of mHealth technology solutions:
  June Kailes, MSW, LCSW, Western University of Health Sciences
- Discussant:
  Mark Bayley, MD, University of Toronto/Toronto Rehabilitation Institute

Frank DeRuyter opened the session by reviewing the rationale for the focus of the SOS Conference on mHealth and mRehab. The rapidly maturing digital health market has shifted dramatically over the past two years from a market focusing on narrowly focused “point solutions” to a broad range of ICT products that are now informing everyday clinical decisions. This is evident in the striking increase of venture capital funding in digital health companies on the forefront of innovation, as well as the impact that ICT is having on the healthcare ecosystem by extending existing care models beyond the hospital or clinic. Still, participants were reminded that despite the advances and convergence of ICT, digital health should complement—not replace—in-person care.

The convergence of technology, medicine, and human activity in mHealth was described by Devin Mann as an enabler that enhances the feedback loop and connections between patients and clinicians. Initially, mHealth was focused on inpatient care and less concerned with outpatient and home care. Today, the spotlight has shifted to outpatient care because of the integration of diverse technologies that allow for the capture and presentation of remote patient data in ways that are useful for clinical decision making. According to Dr. Mann, advances in baseline technologies, medical technology, and societal shifts have enabled this integration. Advances in baseline technologies include improved access (broadband infrastructure, smartphone adoption), improved wireless capabilities, smaller and cheaper monitors and devices, artificial intelligence (AI), machine learning (ML) and natural language processing (NLP), and emerging and improved augmented reality/virtual reality (AR/VR) capabilities. Advances noted in medical technology have influenced the integration of connected electronic health records and patient portals, improved medical devices (pacemakers, insulin pumps), enhanced ability to provide complex care through integrated monitoring in various environments (critical care, post-op, and rehabilitation), and the continued miniaturization of remote monitoring and home diagnostic technologies.

Finally, several societal shifts were identified as enablers of integration. These included changes in family structure that have resulted in less social support and reduced availability of extended family as caregivers, age shifts due to aging baby boomers living longer lives, increasing comfort with asynchronous medical care, increase of chronic disease (obesity, diabetes, and cancer survival), work–life imbalance due to longer work hours and commutes, and changes in consumer standards such as the commodification of healthcare and emphasis on the patient as a consumer. The convergence of these enablers has improved patient–clinician relationships through the shift toward connected care, thus allowing clinicians to view remote data points that were not previously available. Connected data allow for more personalized treatment and better clinician–patient relationships. We want healthcare to be more personalized not less.

David Putrino drew upon his clinical research experience to illustrate the development of innovative technology solutions for individuals in need of better healthcare accessibility. He focused on the importance of technology adoption as a means for demonstrating value to healthcare service delivery organization as well as to clinicians and patients. By way of example, he provided a use-case example of a metropolitan technology-based remote patient monitoring program that was developed in eight sites in White Plains, New York; this program has subsequently been expanded to over 20 sites throughout five states in the US. This program was named the Telehealth Intervention Program for Seniors (TIPS) and utilized remote patient monitoring. It served a senior population with a higher than average percentage over age 65, with most individuals managing chronic conditions and many living in poverty. Prior to implementing the program, health services were costing the county a lot of money. The project included 784 participants who were in the program for at least 12 months. They were provided with a weekly assessment of vital signs and subjective wellness, as well as “wraparound” services [1], to enhance the existing strengths of senior consumers and assist in the development of a network of community and family resources. Participation in the program had a 78.5% compliance rate, an up to 60% reduction in hospital visits in certain high health-risk populations, and an overall 75% reduction in under 30-day readmissions [2].

These unexpected indicators of success of the program resulted from several factors, including social engagement with the young college interns who met with the elderly participants for weekly assessments, raffles, interactions with other older adults, improved health, and the wraparound social support and services. David also discussed some of the research that he has conducted in the field of gamification of rehabilitation for neurological conditions such as stroke. Gaming and overall enjoyment from participating in that research program served as modulators for improved outcomes [3]. Apathy toward the program correlated with poorer outcomes [4].

June Kailes shared her perspective as a consumer with disabilities regarding the user acceptance/adherence and abandonment of mHealth technology solutions. She noted that many people with disabilities have never received rehabilitation services and must rely on their own resources to find and evaluate potential mHealth solutions. June shared and discussed many of the mHealth apps that she has personally used or tried over the past few years. She challenged participants to acknowledge the importance of addressing individual “wish/fix lists” for patients when recommending mHealth solutions and then shared her own personal issues that developers and academics should consider when developing mHealth solutions.

These specific wishes/fixes included the following: communication access/customization (user-defined cues that could push/encourage engagement and ability to adjust auditory output to louder volumes), exportable data so that users—not just the developer—have access to the data, longer battery life to reduce frequency of recharging and risk of running out of charge at inopportune moments, the accuracy of data (e.g., step-counting when hands/arms are not in motion as when pushing a shopping cart), stability (accessibility features often break with updates to apps or operating systems), longevity of mobile apps (many apps and app features simply are not updated or are de-supported), and comparison standards and ratings. Because selecting the right app can be frustrating for people with a disability, there should be a trusted and well researched source that provides accessibility information to consumers.

Finally, Mark Bayley served as discussant to the session. Briefly providing his own perspective as a physician, researcher, and app designer, he suggested that when thinking about barriers to adoption, we need to think about the innovation itself—what are we asking the users to do, who specifically are the users, and what is the nature of the environment where they are going to use the innovation. He emphasized that the technological innovation should complement but not replace clinician-directed care.

Audience members made several comments in response to the observations made by the panelists. It was suggested that, just as developers should partner with consumers to reach desired outcomes, academic researchers/engineers and the private sector should be working together to push mHealth products into the market quicker and with improved user outcomes. Second, a major barrier to knowledge translation and technology transfer is the reluctance of engineers and developers to disseminate their solutions because of uncertainty over who owns the intellectual property or who will get the recognition. Third, it is difficult to scale mHealth technologies. Fourth, there is a lack of convergence between developers and clinicians/users in how they focus on functionality of mHealth technologies. Fifth, clinicians/users tend to focus on why mHealth innovations fail, whereas developers tend to focus on why something works. Finally, it was agreed that while it is difficult to publish failed studies (studies that do not show positive outcomes), the information in such “failed” efforts may be key to developing successful innovations.

**Figure 1:**
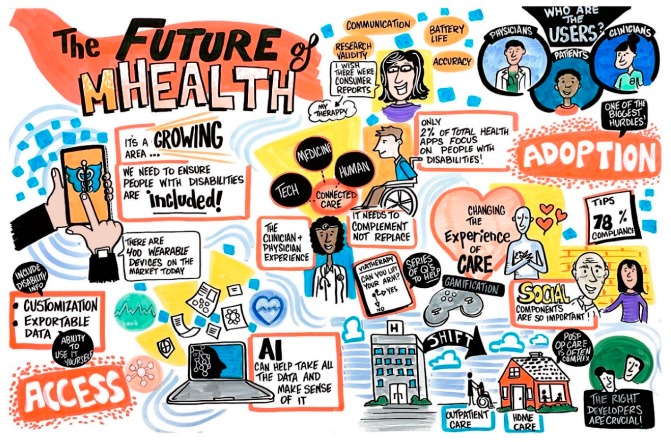
Graphical recording from the session on The Future of mHealth for People with Disabilities.

Figure 1 is the graphical recording that was produced during the session, representing several themes and issues discussed during the session on mHealth.

### 3.2. Session 2: The Future of mRehab for People with Disabilities

- Introduction to mRehab for People with Disabilities:
  Mike Jones, PhD, FACRM, Shepherd Center
- mRehab is the future: the upper limb perspective:
  Catherine Lang, PT, PhD, Washington University
- mRehab: Evaluating mRehab/mHealth mobile apps for usability, engagement, evidence-basis and incorporation of established techniques for behavior change:
  Danielle Jake-Schoffman, PhD, University of Florida
- mRehab: Consumer perspectives, strategies to promote engagement by patients, family, caregivers and other users of mHealth technology solutions:
  Kate Lorig, PhD, Stanford University
- Discussant:
  Paolo Bonato, PhD, Spaulding Rehabilitation, Harvard Medical School

Mike Jones opened the session with a brief overview of factors that have influenced the growth of mRehab, including the growing demand for rehabilitation services in light of shrinking resources available to address the demand; reform in healthcare reimbursement practices and new emphasis on delivering greater value for services; growing acceptance of technology by both providers and consumers of rehabilitation services; the potential of mRehab to address economic, geographic, and logistical barriers that affect participation in outpatient therapy; and the opportunity to improve patient adherence, engagement, and autonomy in home-based rehabilitation.

Using examples from her work in the upper extremity rehabilitation of patients with hemiparesis resulting from stroke, Catherine Lang addressed the challenge of translating improvements in functional capacity that are seen in the outpatient clinic to improvements in functional performance at home and in everyday life. With the advent of mRehab technologies, we can now reliably measure in-home performance of people using wearable sensors (accelerometers). This is exciting and important because it provides the opportunity to demonstrate value by showing that rehabilitation can reduce impairment (a standard clinical outcome measure), improve functional capacity (measured in the outpatient clinic), and improve functional performance in everyday life. However, it is also disconcerting that evidence does not support the assumption that improved capacity translates into improved performance.

She shared preliminary data that were collected from 93 participants in five clinics around the US that compared improvements in the activity observed in the clinic (functional capacity) with activity documented at home; this comparison was done with the use of accelerometers (functional performance). Participants in the study had either stroke or Parkinson’s disease, and sensors measured upper limb activity or walking activity. Preliminary findings have suggested that only about a third of patients who demonstrate a improved capacity in the clinic also demonstrate improved performance at home, whereas another third have shown an improved capacity in the clinic but have not exhibited improved performance at home. While a little less than a third showed no improvement in the clinic or at home, of note was a small portion that exhibited no change in capacity in the clinic but showed an improved performance at home.

These findings highlight the limitations of our current ability to measure performance at home. Constructs that we can reliably measure with sensors include the duration of limb activity, the relative activity of one limb to the other, the intensity of activity, the relative contribution of each limb during bilateral activities, the average magnitude of activity in each limb, and the variation of activity in each limb. However, we cannot currently distinguish between functional and non-functional movements of the limbs, nor can we identify specific activities being performed, compensatory movement patterns (which may reduce the need for increased performance), and perceived ease of movement. Wearable sensor data provide useful insights but also raise additional questions about the best metrics to examine in verifying the benefits of in-clinic therapy to functioning at home. Future sensors and algorithms will be better than current ones and will become critical for research and clinical practice.

Danielle Jake-Schoffman presented strategies for evaluating the usability and efficacy of mobile apps for mHealth and mRehab interventions. While commercial apps are flourishing in health promotion efforts, little evidence exists to support their clinical effectiveness (in promoting desired health behaviors). Part of the problem is the difficulty of using traditional approaches for evaluating efficacy (e.g., randomized clinical trial: RCT). In lieu of clinical trials (which are difficult to conduct with commercial mHealth apps because the apps are often short-lived and go through multiple iterations), researchers have attempted to evaluate app utility through content analysis, usability testing, and non-traditional efficacy studies [5].

Content analysis usually entails interpreting qualitative and text-based material, analyzing information that is in the app or is posted on the app store, and comparing content to clinical guidelines, evidence-based protocols, and known behavior change techniques. Dr. Jake-Schoffman described numerous studies that have used a content analysis approach and which have found that the most commercially available health promotion apps utilize only a handful of evidence-based behavior change techniques.

Usability testing often involves collecting information from targeted users about operability, ease of use, consistency and accuracy, and overall satisfaction with the use of an app. Common evaluation strategies include observations of users under controlled test conditions and real-world use of the app with periodic assessment of users through the use of survey techniques.

Efficacy testing attempts to determine if app use achieves meaningful change in the health behavior of interest, resulting in improved clinical outcomes. Though RCTs are the gold standard for efficacy testing for many areas of health intervention testing, they are time and resource intensive. Dr. Jake-Schoffman suggested an alternative approach that employs the multiphase optimization strategy (MOST) framework. A recent advance in analytic techniques, the MOST approach is an engineering-inspired framework that was proposed by Collins et al. and that allows for the systematic testing of individual intervention components to find an optimized combination [6,7].

The MOST framework may be effectively applied to the development and testing of mHealth and mRehab interventions (e.g., apps) because, once designed, these interventions can be readily delivered, at minimal incremental cost, to a large population of users. Typically delivered via the internet or a stand-alone app, these interventions can also be tailored to individual user preferences or needs. This capacity for tailoring is especially advantageous when applying the MOST framework, which can guide the evaluation of discrete intervention components (e.g., tailored messages) individually and in combination to form a complete intervention.

The MOST framework includes three evaluation phases: (1) preparation, (2) optimization, and (3) evaluation, in which the optimized intervention components are evaluated as a package in a traditional RCT. The MOST approach replaces a series of RCTs. Using this framework, researchers must conduct at least one factorial or micro-randomized trial (or one of several other types of studies) followed by an RCT [8]. The MOST approach can be more accurate and expedient than typical post-hoc analyses at answering questions about which intervention components may work best for which patient populations.

Danielle Jake-Schoffman concluded with a discussion of user engagement as a critical element of the effectiveness of digital behavior change interventions. Though user engagement is generally agreed to be important, there is not a shared understanding of how to usefully conceptualize and measure engagement. She shared a conceptual model that was proposed by Perski, et al. [9] to explain the influencers of engagement with digital behavior change interventions (DBCI); they define engagement as a multidimensional construct consisting of (1) the extent (amount, frequency, duration, and depth) of usage and (2) subjective experience characterized by attention, interest and affect.

The measurable, behavioral dimensions of engagement are grounded in the users’ subjective experience of what it feels like to be engaged with a DBCI. Influencers include contextual factors that are related to the population of users and settings in which they employ an intervention, and factors associated with DBCI content (e.g., specific behavioral intervention techniques and social support features) and delivery (e.g., mode of delivery, control features, message tone and ability to tailor delivery). Using this model, Perski et al., identified four constructs to explain the relationship between engagement and DBCI effectiveness: (1) mechanisms of action (e.g., increased knowledge and skill building), (2) optimal dose (pre-defined level of engagement at which a DBCI is effective), (3) effective intervention elements (e.g., social reinforcement), and (4) unmeasured third—aka intervening—variables that modulate the causal relationship between the intervention and outcome (e.g., beliefs and motivation). Though clearly important to understanding the relationship between engagement and intervention effectiveness, much work is needed to validate these constructs.

**Figure 2:**
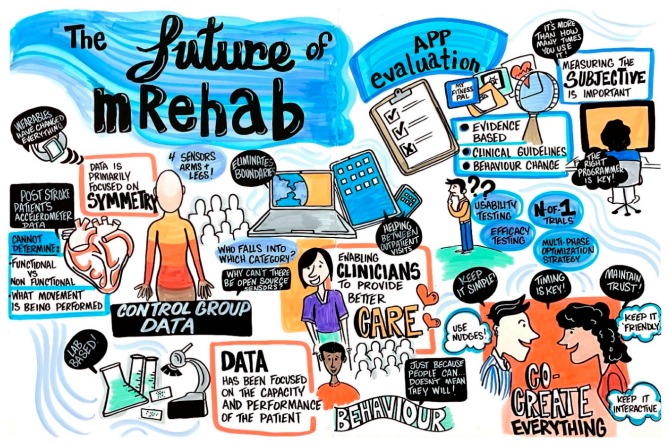
Graphical recording from the session on The Future of mRehab for People with Disabilities.

Kate Lorig drew upon her decades of experience developing, refining, adapting and validating the Chronic Disease Self-Management program [10] to share strategies for engaging end users with disabilities—not only in use of behavior change interventions but also in intervention development; a process she described as co-creation. Co-creation is not limited to gathering user input via interviews and focus groups to gain the endorsement of a pre-formed intervention. Instead, research and development teams must seek user input to identify the “active ingredients” (effective intervention elements) and determine optimal content, dosing, and delivery features. Key tips to engage end-users include: (1) make it easy for them to participate (reduce or eliminate time, technical, and cognitive/mental barriers to participation), (2) establish trust (e.g., tell users what to expect and provide easy access to technical assistance), (3) use nudges and social reinforcement to maintain engagement, and (4) include high levels of interaction among peers to take advantage of social learning opportunities (learning from others like oneself). Figure 2 is the graphical recording that was produced during the session; it represents several themes and issues that were discussed during the session on mRehab.

### 3.3. Session 3: Opportunities and Challenges in mHealth and mRehab

The conference concluded with a two-hour brainstorming session attended by LiveWell RERC staff, invited speakers, and 20 invited participants from Sessions 1 and 2. The key focus of the session was to discuss opportunities and challenges to the growth and adoption of mHealth/mRehab and to establish a consensus among participants concerning the key issues that should drive future research, development, and knowledge translation efforts in the field.

We began with a recap of the salient points made during Sessions 1 and 2, and we proceeded to discuss the scope of the field and necessity of differentiating mHealth and mRehab. As noted earlier, our working definitions of mHealth and mRehab were intended to distinguish between digital health services and supports for self-management of health (mHealth) and the delivery of digital services and supports for home/community-based medical rehabilitation. After much discussion of these and related terms (digital health, connected health) that connote use of ICT to support health and function outcomes, the group reached consensus that mHealth was a more inclusive term and could be used to describe any digital behavior change intervention that was intended to improve health and function outcomes.

Participants were asked to focus on important research needs in the field of mHealth, and discussion focused on the importance of reaching a consensus concerning important process and outcome measures used in research. The engagement framework developed by Perski et al. [9] could prove useful for reaching agreed-upon definitions and measures of adherence, engagement, usability, behavioral intervention elements, and social determinants of health outcomes (typically unmeasured intervening variables that influence the effectiveness of behavior change interventions).

The focus shifted to discussion of development needs in the field, with considerable time devoted to the importance of the customization of user interface features and the choice (and dosing) of intervention elements, as these are the important “active ingredients” of mHealth interventions. Co-creation was viewed as an important strategy for the development of customization features in mHealth applications.

**Figure 3:**
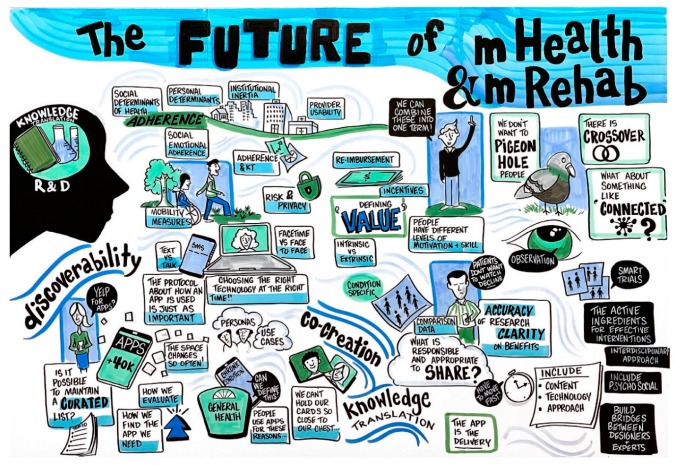
Graphic recording from session on The Future of mHealth and mRehab for People with Disabilities.

Finally, the glaring lack of empirical evidence of the effectiveness of mHealth interventions was acknowledged with a call for more evaluation studies that ideally incorporate recent advances in methodology, such as the MOST framework, to validate the active ingredients of successful mHealth interventions. The session closed with a call for five “big ideas” to advance the field (see also Figure 3):

1. Find and validate the active ingredients of effective mHealth interventions.
2. Engage behavioral scientists in mHealth intervention development to help ensure that known effective behavior-change techniques are included.
3. Incorporate measures of social determinants of health in outcome studies to evaluate mHealth interventions.
4. Include customization and self-tailoring features in both user interfaces and intervention elements in mRehab interventions and technologies. Use co-creation as a development strategy.
5. Focus dissemination and knowledge translation efforts on the discovery of effective mHealth interventions by people with disabilities, caregivers, and clinicians.

## 4. Discussion

The concepts and ideas that were identified and discussed by the invited speakers, participants, and LiveWell RERC staff during the SOS Conference, as well as work conducted over the past four years within the LiveWell RERC, strongly support the continued examination of ICT access that could support independent living and community participation for people with disabilities.

Potential areas of focus include critical social, technical and policy issues, as well as opportunities related to clinical rehabilitation and non-clinical rehabilitation (or “adjacent-clinical”) interventions and technologies. These efforts might include: (1) accessible electronic gaming, esports; (2) the “gamification” (distinct from e-gaming) of rehabilitation technology interventions; (3) exercise and fitness; (4) nutrition and diet; (5) complementary and alternative approaches to healthy living that are related to philosophy of life, psychology and personal training techniques (e.g., spirituality, mindfulness, and meditation; (6) the development of community and peer resources to support education and employment; (7) outreach and education for people with disabilities, caregivers and rehabilitation professionals; (8) extending the reach of rehabilitation technologies and interventions to people in remote areas via broadband technologies that fill the gaps left by national service providers.

Electronic gaming and esports have grown rapidly in popularity in recent years, with consumer spending in the US in 2018 exceeding US$ 43 billion for hardware, software, and accessories (greater than the consumer expenditure on streaming television and music combined) [11], and global audiences of esports events are expected to exceed 640 million people by 2022 [12]. Leading gaming platforms like Xbox produced by Microsoft (Redmond, Washington, WA, USA) include multi-party simultaneous audio and text-based communications that vastly expand options for community participation for people with disabilities. Evidence indicates that the level and quality of community participation are directly related to cognitive, psychological, and physical health and function.

The gamification of technology-based interventions offers potential advantages for user engagement and program adherence. Leveraging the principles of commercial e-gaming (goal setting, the accumulation of points, dashboards for real-time feedback, multiple levels of difficulty, rewards and badges, and in-game communication with other participants), gamification offers the potential to support behavior modification to promote physical activity, healthy lifestyles, medication adherence, and mental health, among other positive behaviors and outcomes [13]. Despite the potential benefits of gamification, more research and development are needed to ensure the incorporation of proper behavior modification principles into the design of technology-based interventions [14] and the durability of positive health outcomes [15].

Exercise/fitness and nutrition/diet represent critical needs for people with disabilities and chronic conditions, but these individuals must overcome (a) accessibility barriers to fitness centers and exercise equipment, (b) the need to adapt exercise and nutrition to the unique physiological needs of people with disabilities, and (c) limited knowledge among trainers, instructors and nutritionists/dieticians. Enhanced access to exercise, fitness, nutrition and diet resources can directly improve individual health, thus enhancing and extending independent living while creating new opportunities for social participation. Technology and social supports can increase education and employment opportunities, both of which enhance independence, personal growth, and economic and psychological well-being.

## 5. Conclusions

The “big ideas” that resulted from the discussion among participants in the SOS Conference should inform the structure and operation of future efforts, including: (1) identifying the active ingredients of interventions, (2) incorporating effective behavior-change techniques (e.g., personal goal-setting, system-generated prompts, and achievement levels and rewards) into all interventions, (3) including measures of social determinants of health in evaluation studies, (4) incorporating user-customizable features into technology solutions and interventions, and (5) ensuring the “discoverability” of LiveWell RERC outputs by people with disabilities, caregivers, and rehabilitation professionals via structured and continuous outreach, education and training.
